# Effect of Magnetized Water on the Stability and Consolidation Compressive Strength of Cement Grout

**DOI:** 10.3390/ma14020275

**Published:** 2021-01-07

**Authors:** Huan-Xiao Hu, Chao Deng

**Affiliations:** 1 School of Geo-Sciences and Info-Physics, Central South University, Changsha 410083, China; hhx@csu.edu.cn; 2 Hunan Key Laboratory of Nonferrous Resources and Geological Hazards Exploration, Changsha 410083, China

**Keywords:** cement grout, magnetized water, stability, compressive strength, magnetization mechanism

## Abstract

In this study, tap water is magnetized by a self-developed device. The conductivity and evaporation of magnetized water (MW) at different temperatures are tested to demonstrate the magnetization mechanism. The results show that the conductivity and evaporation of the magnetized water increase to different degrees compared with regular tap water (RW). The maximum increase in conductivity is 10.6%, and the maximum increase in evaporation is 25.6% and 16.7% at 50 and 80 °C, respectively. Cement grout samples with water-cement ratios (*w/c*) of 0.5 and 1.0 were prepared with magnetized water. The stability of the cement grout mixed with RW and MW under different magnetic conditions is tested. The compressive strength and SEM images of the hardened cement grout samples mixed with RW and MW (under optimum magnetic conditions) are compared. The cement grout prepared with MW has a higher stability compared to that of the control specimen, and the relative change of bleeding volume of the MW cement grout with *w/c* = 0.5 and 1 is 67.69% and 24.36%, respectively. MW has a positive effect on the consolidation compressive strength of cement grout. SEM images show that hardened cement grout with MW has more hydrate crystals, more compact filling space between cement particles, more contact points, and fewer voids compared to that of RW. The influence mechanism of MW on the stability of cement grout is analyzed, which provides a theoretical basis for the application of MW in the field of grouting engineering.

## 1. Introduction

Grouting technology uses hydraulic, pneumatic, or electrochemical methods to inject grouts into the holes, pores, and microfractures of a structure. The injected structure becomes a new structure with high strength, good impermeability, high stability, and strong integrity, which improves the physical and mechanical properties of the injected structure because of the good cementation of the grout. Grouting technology is widely used in the field of civil engineering for applications [1,2] such as tunnel anti-seepage and reinforcement, structure enhancement, foundation treatment, and building lifting and rectification. To meet the different requirements of grouting performance in engineering, a variety of grouting materials with different properties have been produced, among which cement-based grouting materials are the most widely used due to their advantages of good durability, high strength, low cost, and low environmental pollution [3,4,5]. Extensive studies have been carried out, and beneficial achievements have been accomplished for improving the properties of cement-based grouting materials [2,6]. For example, Nguyen et al. [6] pointed out that high stability and fluidity are the basic requirements of cement grout for engineering applications. The improvement in the rheological properties and stability of the grout is the key to improving grouting technology [7]. Furthermore, numerous studies have shown that the properties of cement-based grouts have an important impact on the grouting effect. As reported in [4,8], the cement grout should have high stability because excessive bleeding and settlement instability will lead to incomplete grouting, and the effect of strengthening and anti-seepage will be poor. Similarly, references [9,10] have shown that grout performance is a prerequisite for grouting design and guaranteeing the grouting effect. Therefore, the prerequisite of engineering application is to determine the stability and rheological properties of cement grout.

As a green technology, magnetized water (MW) is widely used in various fields [11,12]; MW can be obtained from water passing through a magnetic field at a certain speed or water that is in a static magnetic field [13,14,15,16,17]. Many studies have shown that magnetized concrete has the advantages of better workability, higher strength, lower cost, and reduced use of chemical additives. Saeid et al. [17,18] reported that the stability and strength of foam concrete were improved from using MW. Majid et al. [19] showed that the self-compacting concrete incorporating MW with various pozzolanic materials has a better working performance and higher strength, especially when the amount of water reducer can be reduced by 45%. Similarly, MW can improve the compressive strength, splitting strength, and elastic modulus of self-compacting concrete, as Hasan et al. [20] noted. In the work [21,22], MW improved the shrinkage cracking resistance, compressive strength, splitting strength, and fluidity of concrete. In addition, it has been pointed out that cement grout in a static magnetic field can accelerate the cement hydration speed, and the structure of hardened cement pastes can be more compact than ordinary cement pastes [23]. Based on the advantages of MW that can significantly improve the working performance and strength of concrete, the changes in the performance of cement-based grout with green MW is a subject that has attracted our attention. To date, no research has been conducted to investigate the effects of MW on the properties of fresh cement grout as grouting materials. In order to better apply the MW in the field of grouting engineering, an experimental investigation was accordingly conducted to fill this research gap.

As mentioned previously, determining the stability and rheological properties of cement grout is a prerequisite for grouting applications, which is also an indispensable part of grouting design and grouting quality control. Therefore, this paper mainly studies the effects of MW under different magnetic conditions on the stability of thin and thick cement grout, and it analyzes the effects of MW on the consolidation compressive strength of cement grout through strength tests and scanning electron microscope (SEM) images. Moreover, combined with the test results of the conductivity and evaporation of MW, the mechanism of the effect of MW on the stability of cement grout is discussed. The effect of MW on the rheological properties of cement grout will be discussed in another research report.

## 2. Experimental Design

### 2.1. Materials

**Figure 1:**
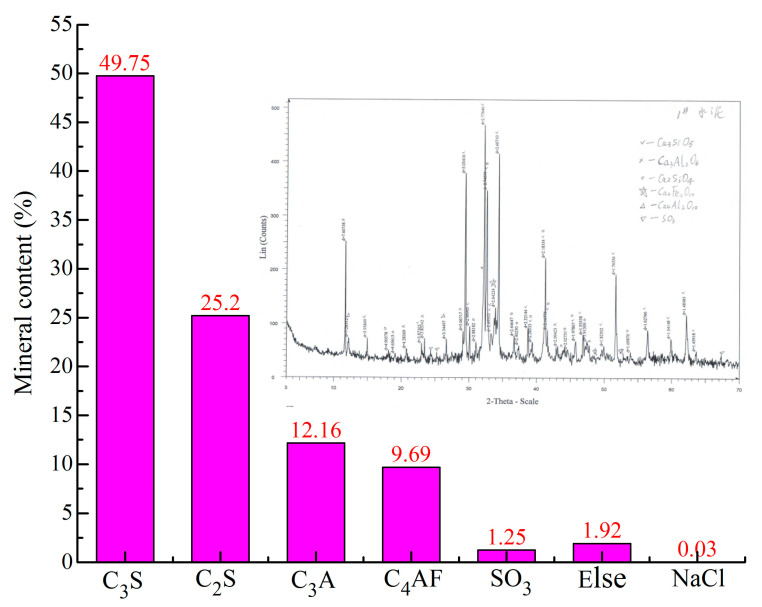
Mineral composition and content of cement.

**Figure 2:**
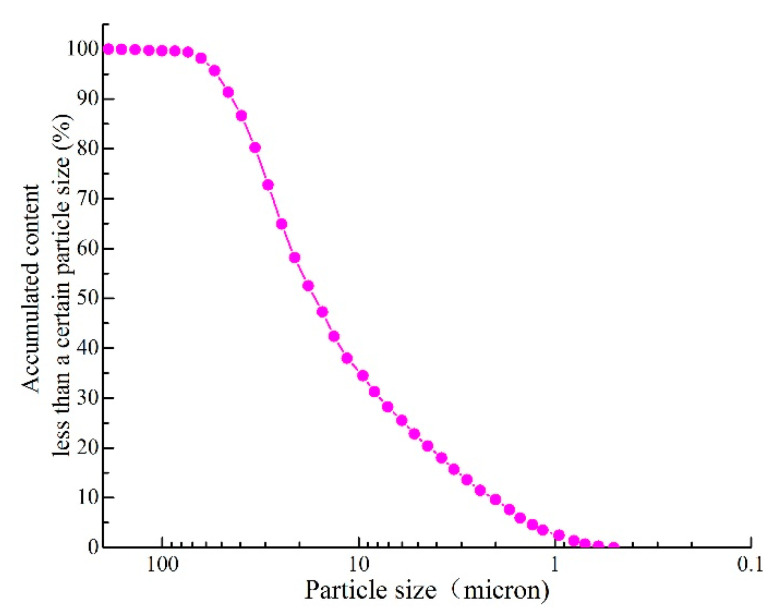
Gradation curve of the cement.

P.O Type 42.5 Portland cement (produced by Nanfang Cement Co., Ltd. Changsha, China) was used throughout this study. The cement is stored in vacuum-sealed bags to ensure that the quality of the cement is not affected by environmental factors. The performance parameters of the cement are shown in Table 1, which are obtained from the factory inspection report. To accurately determine the mineral content and particle size distribution of the cement, the D8-ADVANCE X-ray diffractometer (XRD) (Bruker, Leipzig, Germany) and Malvern-2000 laser particle size analyzer (Malvern, UK) were used for XRD powder diffraction tests and particle size tests. The results are shown in Figure 1 and Figure 2, respectively. The results of the particle size test show that the content of particles larger than 80 µm is less than 1%, and the cement grading is good (uniformity coefficient: *C_u_* = 10.6 and curvature coefficient: *C_c_* = 1.3). The regular tap water (RW) used in the experiment is from a local water supply company (Changsha, China). To ensure the escape of chlorine and a constant temperature, the RW was placed in the temperature control room (setting temperature: 20 °C) for more than 48 h, and the quality of the RW is shown in Table 2.

### 2.2. Magnetization System

**Figure 3:**
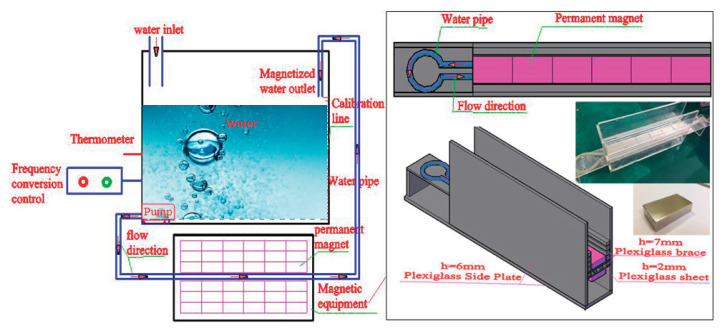
The magnetization system.

According to reviews [11,15], many studies on MW and its applications have shown that the magnetization effect of water is related to the magnetic intensity, and MW can be obtained from uniform, nonuniform, or alternating magnetic fields. As indicated in [24], curing under an alternating magnetic field can improve the compressive strength of fine aggregate concrete by 7.77%. In the application research of cement-based materials with MW, the MW is obtained through a magnetic field at a constant flow speed. The water flow speed, magnetic intensity, cycle times, or magnetization time are used as the magnetization conditions to better study the effects of MW on the performance of cement-based materials [17,18,21,22]. These conditions provided the ideas for the design of the magnetization device in the current study, as shown in Figure 3.

**Figure 4:**
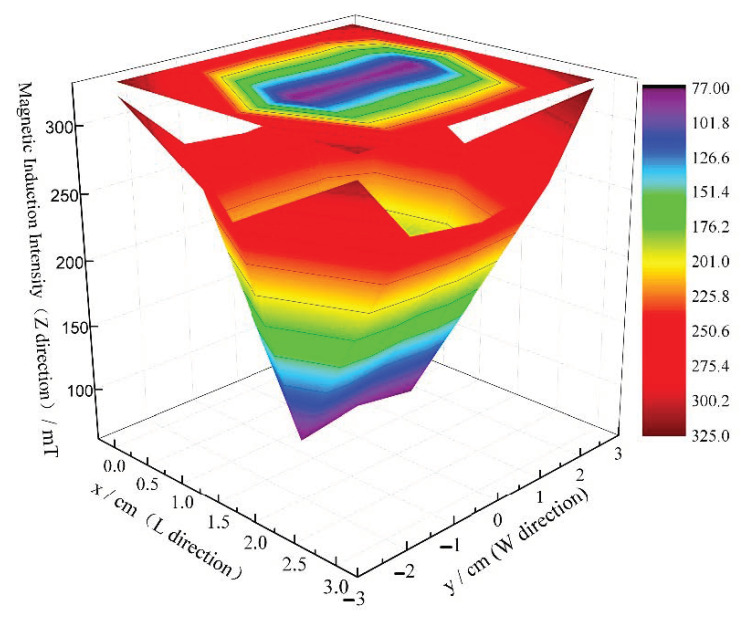
The distribution of magnetic intensity along the thickness direction of the magnet surface.

The main structures of the magnetization device and water pipes are plexiglass and transparent plastic pipes made of diamagnetic materials, respectively. Considering the design concepts of energy conservation and emission reduction, the magnetic field is produced by the combination of rectangular Neodymium magnets (NdFeB). The brand of the permanent magnet is N35 (Changhen magnet, Shanghai, China) with dimensions of 50 mm × 30 mm × 10 mm (length, width, height). Saturation magnetization is conducted along the thickness direction, and an HT-201 high-precision Teslameter (Shanghai Hengtong, Shanghai, China) is used to measure the magnetic intensity. The test results of the magnetic intensity along the thickness direction of a permanent magnet are shown in Figure 4. The magnetic field is obtained by arranging the permanent magnets in a single layer or multilayer with a N-S pole butt joint. The length of the magnetic field has an impact on the performance of cement-based materials. According to [21], the length of the magnetic field of the magnetizing device is designed to be 300 mm. The magnetic intensity can be controlled by increasing the number of superposed layers of the permanent magnet and the height of the magnetic field. The maximum magnetic intensity of the device can reach 758 mT. The water flow speed is controlled by a controllable speed pump and verified by actual measurements.

### 2.3. Magnetization Parameters Design

As mentioned above, the water flow speed, magnetic intensity, cycle times, or magnetization time are used as the magnetization conditions. The parameters for preparing MW include the magnetic intensity, water flow speed, and cycle times, which were selected as the magnetization conditions of MW in this paper. In the work [21,25], the concrete performance is obviously improved under a magnetic strength of 210–270 mT and a water flow speed of 0.65–1.0 m/s. Saeid et al. [17,18] observed a significant improvement in the performance of foam concrete when the magnetic intensity is 650 mT, the water flow speed is 0.75, 1.75, and 2.75 m/s, and cycle times are 5 and 10. In this study, the magnetization parameters are shown in Table 3, and the partial properties of the water under different conditions are measured. Grouting materials with different water/cement ratios (*w/c*) are suitable for different engineering applications. In reference [10], the grouting applications of cement grout with different *w/c* ratios are summarized. The range of the *w/c* ratios for masonry structure repair and rock or soil reinforcement is 0.5–2.0, and the properties of cement grout materials with *w/c* ratios in the range of 1.0–2.0 tend to be stable with the change in the *w/c* ratio [5]. In this paper, the *w/c* ratios of 0.5 and 1.0 represent the low consistency grout and high consistency grout respectively, and the effect of MW on cement grout materials with different consistencies is explored. The cement grout mixed with RW was used as the control mix. The experiment is conducted in a temperature control room (ambient temperature 20 ± 0.5 °C) to reduce the influence of temperature on the performance of the cement grout materials.

### 2.4. Magnetized Water Performance Test

The water conductivity test method is simple, fast, and accurate, and it can be used to verify whether the water magnetization performance changes. A CT3030 conductivity meter (Kedida, Shenzhen, China) was used to test the conductivity of water. It has the functions of automatic temperature compensation and temperature display. One point correction ensures the measurement accuracy when the temperature changes (0–50 °C), the measurement range is 0–1999 ¼s/cm, the resolution is 1 ¼s/cm, and the accuracy is ±2 ¼s/cm +1% FS.

**Figure 5:**
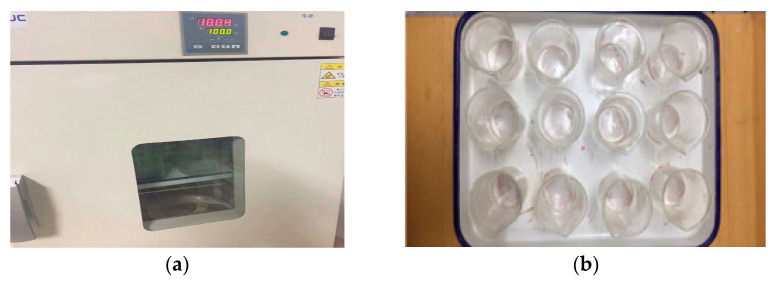
The evaporation test. ((**a**) DHG-9140a, (**b**) The counting cup).

Many scholars have conducted a series of studies on the evaporation of MW [26,27,28], but they all put water in a static magnetic field environment. Although Wang et al. [29] tested the evaporation of MW passing through the magnetic field at a certain flow speed, they focused on the boiling process of MW and did not consider the effect of the boiling point change after magnetization. At present, there is no research on the evaporation of water magnetized at a constant speed through a magnetic field at different temperatures. The evaporation of magnetized and regular water at different temperatures (50 °C, 80 °C) is measured in this study. A constant temperature control drying oven (DHG-9140a, *T_max_* = 250 °C) (Shanghai Yiheng, Shanghai, China) is used in the test, as shown in Figure 5a. The same type of counting cup (diameter: 40 mm and height: 60 mm) is used. The counting cup is small in size and is placed inside the oven. It has a small distribution area, which produces a consistent internal heating environment, as shown in Figure 5b. The water temperature (temperature difference <0.1 °C) is cooled to room temperature in the test. It has been pointed out that the memory effect of MW can last for several hours [11,28], and the influence of the cooling time (5–15 min) of MW can be ignored. The samples are randomly placed in trays, and the positions are evenly distributed. The mean value of the accumulated evaporation (2 h) of the 5 samples is taken as the test result.

### 2.5. Test Procedures and Methods for Cement Grout

**Figure 6:**
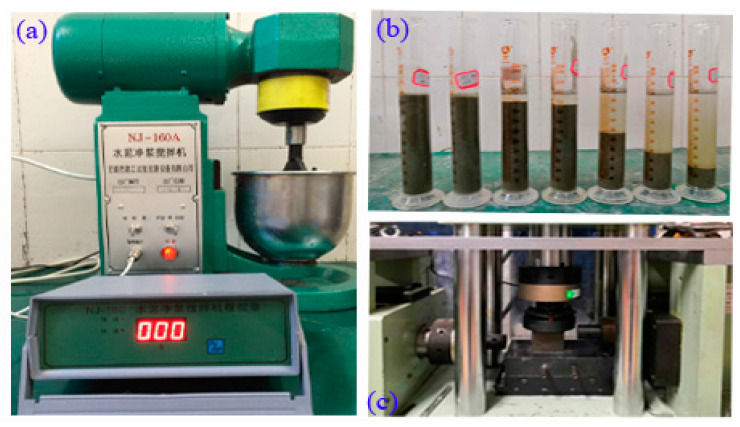
Testing of cement grout ((**a**) The NJ-160A cement paste mixer, (**b**) The bleeding test and (**c**) The compressive strength test).

The NJ-160A cement paste mixer (Nanjing T-Bota Scietech Instruments & Equipment co. ltd, Jiangsu, China) is used for mixing cement grout, as shown in Figure 6a, and the mixing process and technical parameters of the device are shown in Table 4. As mentioned previously, determining the stability of cement grout is a key step for its application. The stability of cement grout refers to the settlement stability of cement grout in a free static state or when the flow speed slows down and there is a loss of control ability under the pressure, i.e., bleeding and water loss. The bleeding test and water-loss test are methods that are commonly used to test the stability of cement grout [1,4]. Bleeding is the process of spontaneous separation of water from cement grout and cement particle settlement. The stability of cement grout can be characterized by the rate of bleeding, which refers to the ratio of the volume of bleeding to the total volume of cement grout. The bleeding rate is an important factor that affects the quality of grouting and is also an important index that reflects the stability of grout and the degree of filling compactness and fullness. In this paper, the stability of the cement grout is studied by the bleeding test, as shown in Figure 6b. The volume of bleeding was recorded every ten minutes for two hours, and the mean value of the 10 samples was taken as the test result. Furthermore, the consolidation compressive strength of cement grout is tested, the section size of specimens is 70.7 mm × 70.7 mm (Chinese standard JGJ/T70-2009), the height of the specimens with *w/c* ratios of 0.5 and 1 are 67 mm and 48 mm (sanding by sandpaper), respectively, as shown in Figure 6c. The effect of MW on the microstructure of hardened cement grout was analyzed by SEM.

## 3. Test Results and Discussion

### 3.1. The Effects of the Magnetic Field on the Water

**Figure 7:**
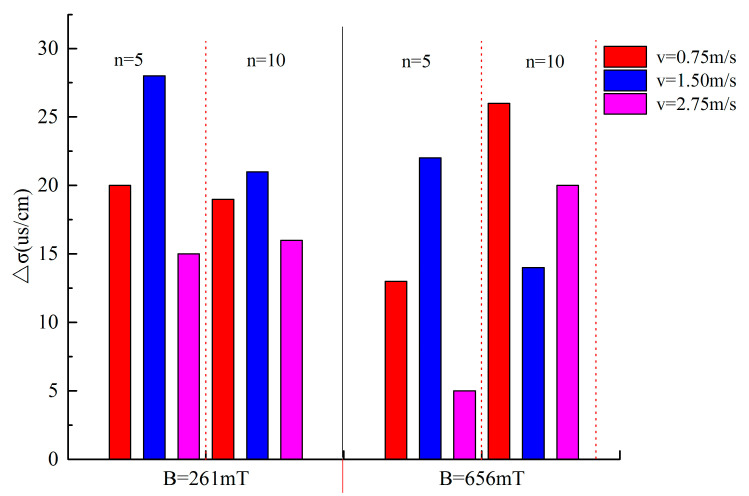
The ∆*σ* of the magnetized water (MW) under different magnetization conditions (temperature: 20 ± 1 °C).

**Figure 8:**
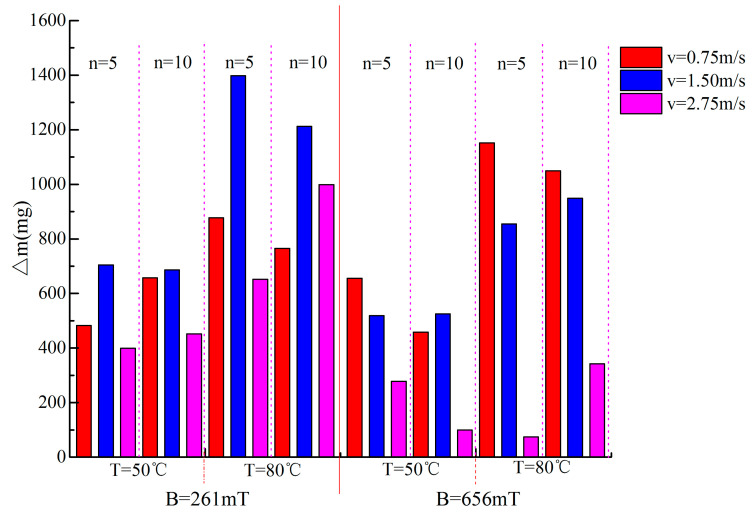
The ∆*m* of the MW under different magnetization conditions (initial water temperature: 20 ± 0.1 °C).

During the performance test of the magnetized tap water, the results for RW were used as the control group to analyze the changes in the conductivity and evaporation of water before and after magnetization—that is, the conductivity change (∆*σ*) is the difference between the conductivity before and after magnetization. Similarly, the evaporation change (∆*m*) is the difference between the accumulated evaporation before and after magnetization, and a positive value represents an increase compared with RW. Figure 7 and Figure 8 show the values of ∆*σ* and ∆*m* for MW under different magnetization conditions. Figure 7 shows that all ∆*σ* are positive, i.e., the conductivity of RW passing through the magnetic field at a constant flow speed increases, and the value of ∆*σ* is different under different magnetization conditions, but it is not strictly monotonous with the magnetization conditions, which is consistent with [11,30,31]. The conductivity of different types of water and aqueous solutions increases under different magnetization conditions; for example, Lee et al. [30] magnetized distilled deionized water and Haitham [31] magnetized several sea water solutions, and the conductivity increased significantly. However, some studies have shown that the conductivity of water and aqueous solutions decreased after magnetic treatment; more details can be found in [11]. Whether the conductivity increases or decreases after the magnetic treatment, this change indicates that the electrical property of the water has changed due to the magnetic field, so it is possible to judge whether the water and its aqueous solution are magnetized by the conductivity test.

Figure 8 shows that the values of ∆*m* at different ambient temperatures (50 °C, 80 °C) after magnetization are positive; that is, the cumulative evaporation of the magnetized tap water increases, but it is not strictly monotonous with the magnetization conditions, and the result is similar to [15,26,27,32]. In particular, when the magnetic intensity *B* = 261 mT, cycle times *n* = 5 and water flow speed *v* = 1.5 m/s, the values of ∆*m* at the ambient temperatures of 50 °C or 80 °C are the largest, with growth rates of 25.6% and 16.7% compared to the control specimens, respectively. Similarly, ∆*σ* has similar characteristics (Figure 7), with a maximum of 10.6%, and the characteristics disappear when the magnetic intensity is large (*B* = 656 mT); a similar result was also reported by other researchers [21,29].

**Figure 9:**
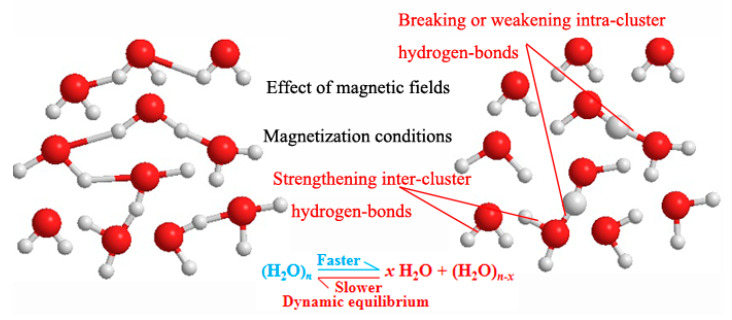
The effect of the magnetic field on water.

Liquid water in nature exists in the form of a few single water molecules and a large number of water molecular clusters based on hydrogen bonding [17]. It is well known that Brownian motion is continually occurring all the time in liquid water. Water evaporation is a process in which a single water molecule is separated from liquid water. The more single water molecules and small water clusters there are (the more active the water is), the more intense the Brownian motion, and the faster the water evaporates, that is, the evaporation of water per unit time increases [27]. Water has conductivity because water molecules decompose impurities into positive and negative ions producing a weak ionization of water. The greater the activity of water is, the stronger the affinity for ions in water, which enhances the ionization of water and decomposition of impurities into positive and negative ions and improves the conductivity of liquid water [33]. The results in Figure 7 and Figure 8 show that the number of single water molecules or small water molecular clusters increases after water passes through the magnetic field at a constant flow speed, which is consistent with the popular explanation of the magnetization mechanism of water [27,34,35]. The water activity is enhanced after magnetization, and the effect of the magnetic field on water is shown in Figure 9, and this result is a simulation based on the analysis of experimental results.

### 3.2. Effect of Magnetized Water on the Stability of Cement Grout

**Figure 10:**
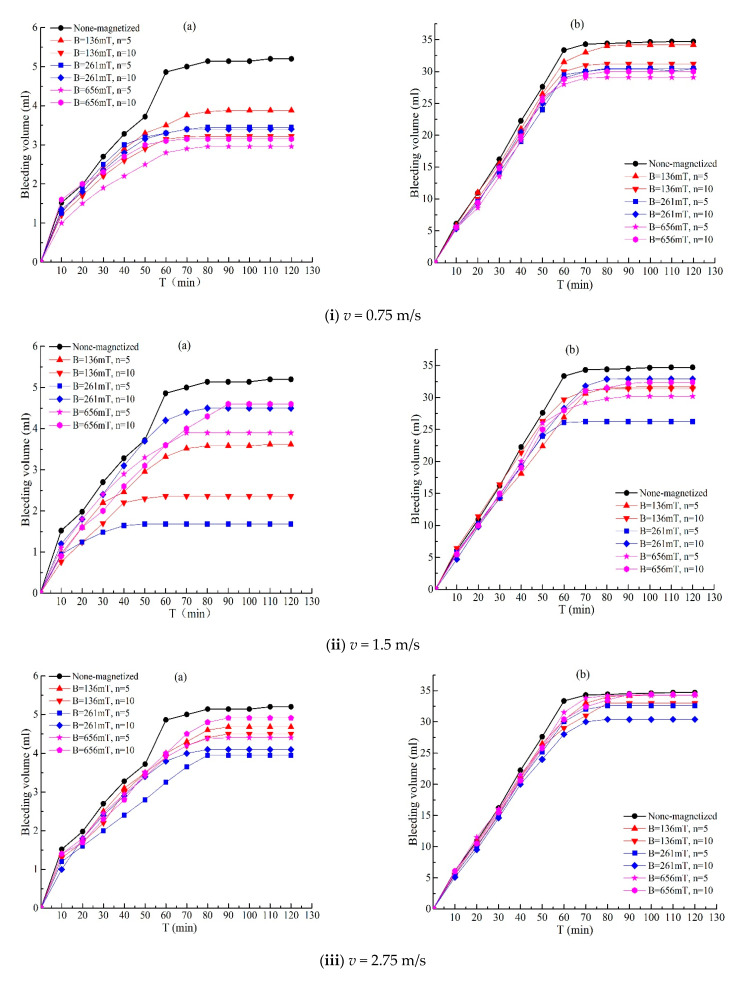
Bleeding volume of cement grouts under different magnetic conditions (environment temperature 20 ± 1 °C, (**a**) water-cement ratios (*w/c*) ratio 0.5:1, (**b**) *w/c* ratio 1:1).

The stability of cement grout mixed with MW is shown in Figure 10. Each point in Figure 10 is the mean value of ten independent test results. As shown in Figure 10, the bleeding curve of cement grout is composed of a fast increasing stage, a slow increasing stage, and a basic stable stage. The average slope value of the increase stage (fast and slow stage) reflects the bleeding speed of cement grout. The lower slope value means that the water bleeding speed of cement grout is slower, that is, the stability of the cement grout is better. A similar analysis can be seen in [17,18]. At the same time, the stability of cement grout with a short basic stabilization time and less bleeding volume is higher than that of cement grout with a long basic stabilization time and more bleeding volume. The average slope value of the bleeding curve of cement grout mixed with MW is lower than that of the control specimens, the basic stabilization time is shorter, and the bleeding volume is less; that is, MW can improve the stability of cement grout, as shown in Figure 10.

When cement and water are mixed to form cement grout, the minerals in the cement will be hydrated and dissolve, and the grout has fluidity and a certain plasticity at the initial time. The sinking speed of various solid particles in the mixed cement grout is different (some even float up), which causes the relative displacement of each particle, so the distribution of the solid particles is uneven, and even the structure of the cement grout is layered, which seriously damages the overall uniformity of cement grout after consolidation. Assuming that the solid particles are spheres, the forces acting on the solid particles in the cement grout are analyzed from the perspective of mechanics. There are three kinds of forces that act on solid particles: the particle weight, grout viscous resistance, and buoyancy. According to Stokes Law, the kinetic equation can be expressed as:

(1) $$\frac{4}{3}\pi r^{3}\rho g-6\pi r\mu V-\frac{4}{3}\pi r^{3}\rho_{c}g=\frac{4}{3}\pi r^{3}\rho \frac{dV}{dt}$$

where *ρ* and *ρ_c_* are the solid particle density and grout density, respectively, kg/m^3^; *μ* is the viscosity coefficient of grout, N·s/m^2^; *V* is the particle movement speed, m/s; and *r* is the solid particle radius. According to Stokes law, the settlement speed *V* can be expressed as:

(2) $$V=\frac{2r^{2}g(\rho -\rho_{c})}{9\mu }.$$

According to Formula (2), the settlement speed *V* is directly proportional to the solid particle radius *r* squared and the density difference between the solid particle and grout, while it is inversely proportional to the viscosity coefficient *μ*. As mentioned before, MW has more single water molecules and small water molecular clusters than of RW, and the water film on the surface of the solid particles is relatively thin [22,36]. The thinner water film between the solid particles causes the viscosity coefficient *μ* of cement grout to increase. Due to mechanical effects such as grinding, a large number of micro cracks are inevitably produced on the surface of cement particles, which are invisible to the naked eye. Single water molecules and small water molecule clusters are more likely to enter into the cracks of cement particles to produce a hydration reaction, which causes the cement particles to decompose into smaller cement particles. The r of the solid particles in cement grout mixed with MW decreases. It can be seen from the above analysis that the settlement speed of the solid particles in cement grout mixed with MW is slow, and the bleeding speed is slow. More single water molecules and small water molecular clusters participate in the hydration reaction of the cement minerals, which leads to a decrease in the bleeding volume compared to that of RW.

Figure 10 also shows that the bleeding volumes of the cement grouts mixed with MW under different magnetization conditions are different, which indicates that the water magnetized with different magnetization conditions has different effects on the stability of cement grout. In this study, the effect of MW on the stability of cement grout under different magnetization conditions is compared by using the bleeding ratio *β*, relative change rate *η* of the bleeding volume, and change rate ∆*η* of *η* with the cycle times as the quantitative indexes, which are as follows:

(3) $$\beta =V_{w}/V_{c}$$

(4) $$\eta =\frac{V_{RW}-V_{MW}}{V_{RW}}\times 100\%$$

(5) $$\Delta \eta =\eta_{10}-\eta_{5}$$

where *V_w_* represents the bleeding volume of cement grout and *V_c_* is the volume of cement grout, which is 100 mL. *V_RW_* and *V_MW_* represent the bleeding volume of cement grout mixed with RW and MW, respectively. *η_10_* and *η_5_* represent the *η* when the cycle times are *n* = 5 and *n* = 10, respectively. Obviously, *β* reflects the size of the bleeding volume of cement grout, a low *β* value indicates that the stability of cement grout is high. When *η* is positive, MW has a positive effect on the stability of the cement grout, and a higher *η* value indicates that the stability of the grout is higher. A positive value for ∆*η* indicates that the stability of cement grout at *n* = 10 is higher than that at *n* = 5; otherwise, it is lower. The statistical results of the bleeding test are shown in Table 5. The standard deviation in the table is the statistical result of ten samples.

The *β* values of cement grout mixed with MW under different magnetization conditions are lower than that of RW at the same *w/c* ratio, and *η* is positive, as shown in Table 5. This also demonstrates that the RW passing through the magnetic field at a constant flow speed has a positive effect on the stability of the cement grout. The *η* values are different with the change in the magnetic induction intensity, water flow speed, and cycle times. Under the magnetization condition of *B* = 261 mT, water flow speed *v* = 1.5 m/s, and cycle times *n* = 5, the stability of the cement grout has the greatest improvement at a *w/c* ratio of 0.5 and 1, and the *η* values are 67.69% and 24.36%, respectively. It is worth noting that the value of *η* for cement grout mixed with MW at a *w/c* ratio of 0.5 is significantly higher than that at a *w/c* ratio of 1, which indicates that MW significantly improves the stability of cement grout with a high consistency. A possible explanation for this is that the content of cement particles in the cement grout with a high consistency is relatively large, there are more single water molecules and small water molecules in MW that can participate in the cement hydration reaction. For cement grout with a low consistency, the water content is large, which will produce more surplus water and relatively increase the bleeding.

The values of ∆*η* are all positive values when the magnetic intensity is low (*B* = 136 mT), which can be found from Table 3. However, when *B* is 261 mT, the increase in the cycle times is beneficial to the stability of the cement grout only when the water flow speed is 0.75 m/s. The values of ∆*η* are negative in other cases, which is more obvious when *B* is 656 mT. This illustrates that we can try to increase the cycle times to improve the stability of the cement grout when the value of *B* is low. In contrast, the increase in cycle times has a negative effect on the stability when the magnetic intensity is too large. This may be due to the change in the dynamic equilibrium of the hydrogen bonds between the water molecules, as shown in Figure 9; more details can be found in [34,35]. When the magnetic intensity is too large and the cycle times (effective magnetization time) increase, the dynamic equilibrium of water tends to slowly strengthen the hydrogen bond between water molecules. Therefore, when the field strength is appropriate and the effective magnetization time increases, the hydrogen bond between the water molecules can be weakened or broken, which improves the stability of cement grout. When the field strength is large, the magnetization time is too long, resulting in the phenomenon of over-magnetization, which is not conducive to improving the stability of cement grout; this explanation is consistent with that of Saeid et al. [17,18]. In the application of MW, over-magnetization should be prevented to ensure the best effect of the MW.

**Figure 11:**
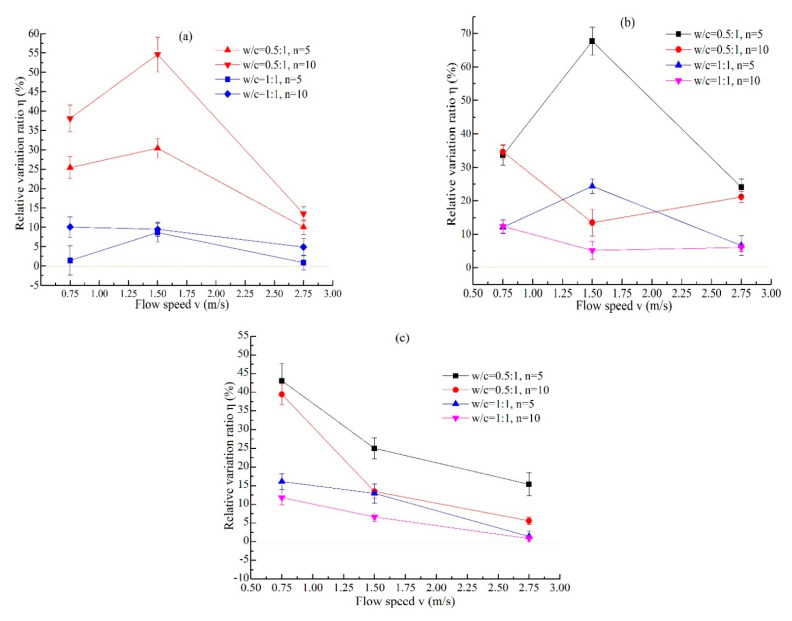
The values of *η* change with water flow speed under different magnetization conditions ((**a**) *B* = 136 mT, (**b**) *B* = 236 mT, (**c**) *B* = 656 mT).

**Figure 12:**
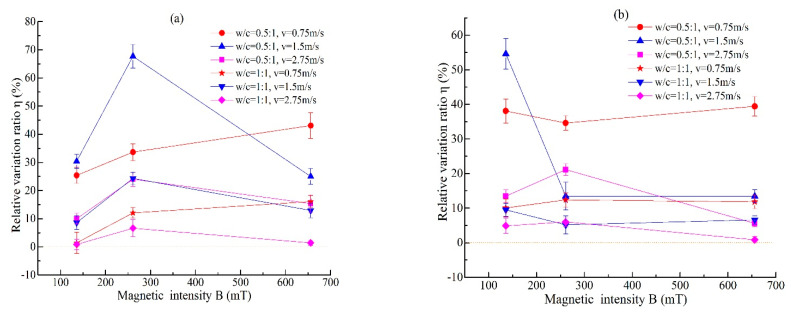
The values of *η* change with magnetic intensity under different magnetization conditions ((**a**) *n* = 5, (**b**) *n* = 10).

The water that is magnetized by passing through a magnetic field is different from the MW obtained by placing water in a magnetic field, and the water flow speed also affects the magnetization effect. The study in [16] also states that the magnetic effect of flowing water is more significant than that of water that is not flowing. The values of *η* under different magnetization conditions change with the water flow speed and magnetic intensity, as shown in Figure 11 and Figure 12, respectively. The trends under different *w/c* ratios are basically the same. When the magnetic intensity is 136 mT, *η* reaches its maximum value at *v* = 1.5 m/s, only when the *w/c* ratio is 1, the value of cycle times is *n* = 5, and the *η* value at *v* = 1.5 m/s is slightly less than that at *v* = 0.75 m/s, as shown in Figure 11a. When the magnetic intensity is 261 mT, the value of cycle times is *n* = 5; the *η* values increase first and then decrease with the water flow speed, and the maximum value is obtained at 1.5 m/s, which is similar to the trend of *B* = 136 mT. The change trend of *n* = 10 is opposite that of *n* = 5, and *η* reaches its maximum value at *v* = 0.75 m/s, as shown in Figure 11b. When the magnetic intensity is 656 mT, *η* decreases with increasing water flow speed, and the maximum value is obtained at 0.75 m/s. This shows that the increase in flow speed is not conducive to the improvement of stability in the cement grout when the field strength is large. At the number of cycle times *n* = 5, the values of *η* increase with increasing of magnetic intensity when the water flow speed is *v* = 0.75 m/s. However, when the water flow speed of water is larger (*v* = 1.5 m/s and 2.75 m/s), the values of *η* first increase and then decrease, as shown in Figure 12a. At the number of cycle times *n* = 10 (Figure 12b), the law of *η* changing with the magnetic intensity is not obvious, and it varies with the water flow speed. Finally, it can be concluded that MW has a positive effect on the stability of cement grout. However, the improvement in the stability depends on the field strength, water flow speed, and cycle times. In the application of MW, over-magnetization should be avoided, and the best magnetization conditions should be determined to obtain the best results.

### 3.3. The Consolidation Compressive Strength and SEM Images of Cement Grout

**Figure 13:**
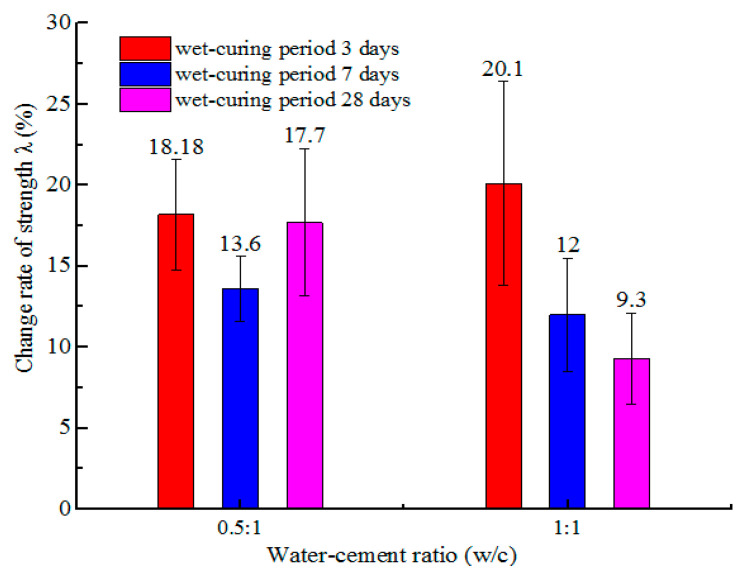
The values of *λ* of hardened cement grouts.

**Figure 14:**
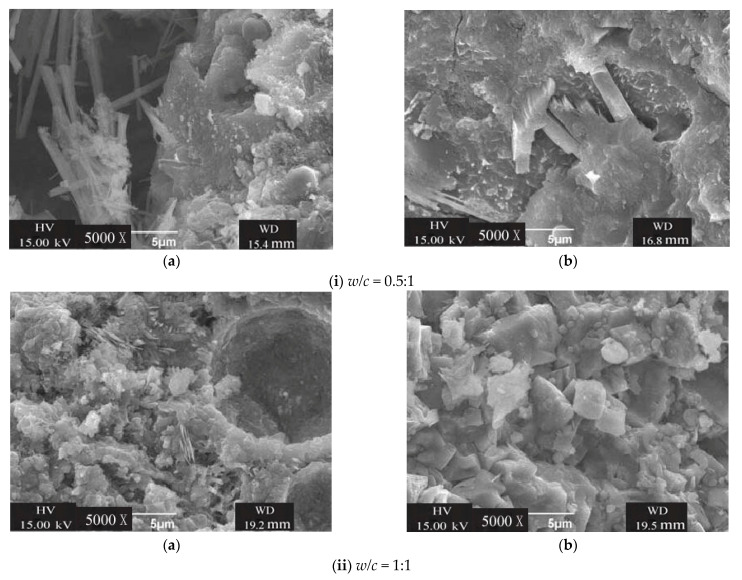
The SEM images of hardened cement grouts (**a**) RW, (**b**) MW.

In the application of grouting technology, the cement grout should have a high stability, and other factors such as the consolidation compressive strength of cement grout should be considered. As described in Section 3.2, *B* = 261 mT, *v* = 1.5 m/s, *n* = 5 are selected as the best magnetization conditions to study the effect of MW on the compressive strength and microstructure of hardened cement grout. The control specimen is hardened cement grout mixed with RW. The change rates of compressive strength *λ* of hardened cement grout mixed with MW and RW are compared, and the curing ages of the test blocks are 3, 7, and 28 days, as shown in Figure 13. The SEM images of hardened cement grout at 28 days are shown in Figure 14. The change rate of the compressive strength *λ* can be expressed as:

(6) $$\lambda =\frac{\sigma_{MW}-\sigma_{RW}}{\sigma_{RW}}\times 100\%$$

where *σ_MW_* and *σ_RW_* represent the compressive strength of hardened cement grout mixed with MW and RW, respectively, which are the mean values of the compressive strength tests of the ten specimens, MPa. A positive value for *λ* indicates that the compressive strength of the hardened cement grout mixed with MW increases; otherwise, it decreases.

The values of *λ* for hardened cement grout mixed with MW at curing ages of 3, 7, and 28 days are positive, which means that MW has a positive effect on the compressive strength of hardened cement grout. At a curing age of 3 days, the values of *λ* for hardened cement grout with *w/c* ratios of 0.5 and 1.0 are a maximum, reaching 18.18% and 20.1%, respectively, which indicates that MW has the most beneficial effect on improving the early compressive strength of hardened cement grout. This may be attributed to the high activity obtained by tap water passing through the magnetic field at a constant speed, as the MW has more single water molecules and small water molecular clusters than that of RW. The single water molecules and small water molecular clusters available for the hydration process may accelerate the hydration rate, which can lead to an increase in the compressive strength of hardened cement grout. The SEM images of the hardened cement grout at curing ages of 28 days show that there are more hydrate crystals in hardened cement grout mixed with MW, the filling of the space between the cement particles is denser, the gap is increasingly smaller, and there are many contact points than that of RW. Therefore, the compressive strength of hardened cement grout mixed with MW is higher than that of the control specimens, which is similar to the conclusion of many studies on MW concrete [17,18,21,22], as shown in Figure 14.

## 4. Conclusions

In this study, the conductivity of magnetized water and its evaporation at different temperatures are studied to demonstrate the magnetization mechanism. The effect of magnetized water on the stability and consolidation compressive strength of cement grout has been studied, and the following conclusions can be drawn:

1. The magnetic field can change the physical and electrical properties of tap water, and the evaporation and conductivity of magnetized tap water are increased. At ambient temperatures of 50 and 80 °C, the maximum increase in the accumulated evaporation at 2 h was 25.6% and 16.7%, respectively, and the maximum increase in conductivity was 10.6%;
2. Compared with the cement grout with regular tap water, the cement grout mixed with magnetized water had a slower bleeding speed and smaller bleeding volume;
3. The magnetic intensity, water flow speed, and cycle times are important factors in the stability of cement grout. In the application of magnetized water, over-magnetization should be avoided, and the best magnetization conditions should be determined to obtain the best results;
4. The cement grout mixed with magnetized water under the condition of *B* = 261 mT, *v* = 1.5 m/s, and *n* = 5 obtained the most beneficial stability of the cement grout, and the stability of the cement grout with *w/c* ratios of 0.5 and 1 increased by approximately 67.69% and 24.36% relative to the control specimens, respectively;
5. Magnetized water has a positive effect on the consolidation compressive strength of cement grout, which is beneficial for improving the early consolidation compressive strength of cement grout. The consolidation compressive strength of cement grout with *w/c* ratios of 0.5 and 1 increased by about 18.8% and 20.1% under the best magnetization conditions, respectively;
6. The SEM images showed that there are more hydrate crystals in hardened cement grout mixed with MW, the filling of the space between the cement particles is denser, the gap is increasingly smaller, which led to a significant improvement in the microstructure compared with that of the control specimens.

Magnetized water has a positive effect on the stability of cement grout under normal pressure, the improvement in the stability depends on the field strength, water flow speed, and cycle times; this will support our further research. The stability of cement grout with magnetized water under pressure condition (the force of grouting) should be explored in future research steps.

## Figures and Tables

**Table 1:** Performance parameters of P.O 42.5 Portland cement.

Item	Specific Surface Area (m^2^/Kg)	Loss on Ignition (%)	Slag Content (%)	Alcoholamine Grinding Aids (%)	Initial Setting Time (min)	Final Setting Time (min)	Alkali Content (%)	Soundness
P.O 42.5	356	2.76	9.5	0.1	176	335	0.4	Qualified
Standard	≥300	≤5.0	5.0~20.0	≤0.5	≥45	≤600	0.6	Qualified

Note: the standard is the Chinese standard.

**Table 2:** The quality of the regular tap water (RW).

Item	Turbidity (NTU)	Free Residual Chlorine (mg/L)	Total Hardness (CaCO_3_)	Oxygen Consumption (mg/L)	PH	Iron Content (mg/L)	Manganese Content (mg/L)	Aluminum Content (mg/L)	Colority
Test value	0.16	0.18	107	0.99	7.14	<0.05	<0.05	0.035	<5
Standard	<3	0.3~4	<450	<5	6.5~8.5	<0.3	<0.1	<0.2	<15

Note: Oxygen consumption was calculated by O_2_ using CODMn method; Lead content <0.004 (mg/L); Cadmium content <0.0002 (mg/L); Mercury content <0.0001 (mg/L); Selenium content <0.001 (mg/L); Arsenic content: 0.001 (mg/L). The standard is the Chinese standard.

**Table 3:** Design of experimental parameters.

Magnetic Intensity *B* (mT)	Water Flow Speed *v* (m/s)	Cycle Times *n*	Water/Cement Ratio (*w/c*)	Water Performance Test/Sample Quantity	Grout Performance Test/Sample Quantity
0	0	0	0.5/1.0	Yes/5	Yes/10
136	0.75	5/10		No	
	1.5			No	
	2.75			No	
261	0.75			Yes/5	
	1.5			Yes/5	
	2.75			Yes/5	
656	0.75			Yes/5	
	1.5			Yes/5	
	2.75			Yes/5	

**Table 4:** The mixing process and technical parameters of NJ-160A.

Width of Mixing Blade (mm)	Size of Mixing Pot (mm)			Revolution Speed (r/min)		Rotation Speed (r/min)		Automatic Control Time (s)		
	Inner Diameter	Depth	Wall Thickness	High Speed	Low Speed	High Speed	Low Speed	High Speed	Stop	Low Speed
111	160	140	1	125 ± 10	62 ± 5	285 ± 10	140 ± 5	120 ± 5	15	120 ± 3

**Table 5:** The statistical result of test sample.

Magnetic Intensity (mT)	*w/c*	Flow Speed (m/s)	Cycle Times	Bleeding Ratio *β* (%)	Standard Deviation	Relative Variation Ratio *η* (%)	Increment of *η* by Cycle Times ∆*η* (%)
0	0.5	0	0	5.20	0.5099	0.00	
	1			34.69	0.7392	0.00	
136	0.5	0.75	5	3.88	0.6632	25.38	+12.7
			10	3.22	0.4952	38.08	
		1.5	5	3.62	0.4578	30.38	+24.23
			10	2.36	0.4454	54.61	
		2.75	5	4.68	0.3889	10.00	+3.46
			10	4.50	0.8112	13.46	
	1	0.75	5	34.20	0.7785	1.41	+8.65
			10	31.21	0.5999	10.06	
		1.5	5	31.70	1.4236	8.62	+0.86
			10	31.41	0.8602	9.48	
		2.75	5	34.40	0.8788	0.84	+4.03
			10	33.00	0.4232	4.87	
261	0.5	0.75	5	3.45	0.3631	33.65	+0.95
			10	3.40	0.8784	34.62	
		1.5	5	1.68	0.4833	67.69	−54.23
			10	4.50	0.1833	13.46	
		2.75	5	3.95	0.4950	24.04	−2.89
			10	4.10	0.1561	21.15	
	1	0.75	5	30.50	0.3890	12.08	+0.28
			10	30.41	0.7411	12.36	
		1.5	5	26.24	0.7419	24.36	−19.20
			10	32.90	0.4899	5.16	
		2.75	5	32.40	0.5680	6.60	−0.60
			10	32.61	0.2986	6.00	
656	0.5	0.75	5	2.96	0.6662	43.08	−3.66
			10	3.15	0.3232	39.42	
		1.5	5	3.90	0.6777	25.00	−11.54
			10	4.50	0.4511	13.46	
		2.75	5	4.40	0.7996	15.38	−9.81
			10	4.91	0.3665	5.57	
	1	0.75	5	29.10	0.3222	16.11	−4.32
			10	30.60	0.3692	11.79	
		1.5	5	30.20	0.4560	12.94	−6.34
			10	32.40	0.7996	6.60	
		2.75	5	34.20	0.8798	1.41	−0.57
			10	34.40	0.4848	0.84	

## Data Availability

The data used to support the findings of this study are available from the paper.
